# Prediction Models for Radiation-Induced Neurocognitive Decline in Adult Patients With Primary or Secondary Brain Tumors: A Systematic Review

**DOI:** 10.3389/fpsyg.2022.853472

**Published:** 2022-03-31

**Authors:** Fariba Tohidinezhad, Dario Di Perri, Catharina M. L. Zegers, Jeanette Dijkstra, Monique Anten, Andre Dekker, Wouter Van Elmpt, Daniëlle B. P. Eekers, Alberto Traverso

**Affiliations:** ^1^Department of Radiation Oncology (Maastro Clinic), School for Oncology and Developmental Biology (GROW), Maastricht University Medical Center, Maastricht, Netherlands; ^2^Department of Radiation Oncology, Cliniques Universitaires Saint-Luc, Brussels, Belgium; ^3^Department of Medical Psychology, School for Mental Health and Neurosciences (MHeNS), Maastricht University Medical Center, Maastricht, Netherlands; ^4^Department of Neurology, School for Mental Health and Neuroscience (MHeNS), Maastricht University Medical Center, Maastricht, Netherlands

**Keywords:** cranial irradiation, cognitive dysfunction, neurotoxicity, machine learning, artificial intelligence

## Abstract

**Purpose:**

Although an increasing body of literature suggests a relationship between brain irradiation and deterioration of neurocognitive function, it remains as the standard therapeutic and prophylactic modality in patients with brain tumors. This review was aimed to abstract and evaluate the prediction models for radiation-induced neurocognitive decline in patients with primary or secondary brain tumors.

**Methods:**

MEDLINE was searched on October 31, 2021 for publications containing relevant truncation and MeSH terms related to “radiotherapy,” “brain,” “prediction model,” and “neurocognitive impairments.” Risk of bias was assessed using the Prediction model Risk Of Bias ASsessment Tool.

**Results:**

Of 3,580 studies reviewed, 23 prediction models were identified. Age, tumor location, education level, baseline neurocognitive score, and radiation dose to the hippocampus were the most common predictors in the models. The Hopkins verbal learning (*n* = 7) and the trail making tests (*n* = 4) were the most frequent outcome assessment tools. All studies used regression (*n* = 14 linear, *n* = 8 logistic, and *n* = 4 Cox) as machine learning method. All models were judged to have a high risk of bias mainly due to issues in the analysis.

**Conclusion:**

Existing models have limited quality and are at high risk of bias. Following recommendations are outlined in this review to improve future models: developing cognitive assessment instruments taking into account the peculiar traits of the different brain tumors and radiation modalities; adherence to model development and validation guidelines; careful choice of candidate predictors according to the literature and domain expert consensus; and considering radiation dose to brain substructures as they can provide important information on specific neurocognitive impairments.

## Introduction

Brain tumors refers to two general types: primary malignant tumors accounting for 1% of the newly diagnosed cancer patients and secondary/metastatic brain tumors occurring in 20% of the cancer patients (Siegel et al., 2018; Sacks and Rahman, 2020). Patients with primary or metastatic brain tumors are characterized by complex and sometimes severe symptoms, usually associated with poor prognosis. Radiation Therapy (RT) is an indispensable therapeutic and prophylactic component for extending patient survival as well as effective symptom relief (Grunert et al., 2018). Depending on the location of the tumor, the use of brain RT has been confounded by the challenge of damaging critical vascular and neural structures. Patients treated with RT to the brain might experience acute irradiation triggered inflammation and be at risk for late toxicity sequelae (Tanguturi and Alexander, 2018). One of the possible side effects of RT is neurocognitive decline.

Neurocognitive decline is a progressive and often disabling side effect reported in 50–90% of the patients who receive whole brain irradiation (Pazzaglia et al., 2020). The literature suggests that radiation-induced neurocognitive decline includes damage in multiple neural cell types, increasing neuroinflammation, reducing neurogenesis in the hippocampus, and causing functional and structural alterations in the brain blood vessels (Makale et al., 2017). Major neurocognitive deficits including, dysfunctions related to learning, attention, memory, processing speed, spatial processing, and executive capabilities may become manifest from months to years after irradiation (McDuff et al., 2013; Michaelidesová et al., 2019). Improvements in radiation delivery technologies (e.g., stereotactic radiotherapy, intensity modulated radiotherapy, and proton beam therapy) allow reducing the dose delivered to the normal brain tissue (Scaringi et al., 2018; Weber et al., 2020). Identification of the patients who might benefit from a certain treatment, will increase efficacy and potentially reduce costs.

Clinical modeling refers to the use of mathematical equations to support physicians in proposing individualized treatment indications (Chen, 2020). Although medical literature overflows with articles offering to help clinicians and patients in decision making, front-line clinical use of the available prediction models remain underutilized mainly due to lack of adherence to model development and evaluation guidelines (Chowdhury and Turin, 2020). The following considerations are crucial to build a reliable prediction model: obtaining high quality multidimensional data from patients who represent the intended target population, including easy-to-use predictors which have been measured without knowledge of the outcome data, using standard outcome definition with reasonable time interval since predictor assessment, and handling statistical concerns and complexities during analysis with appropriate performance assessment (Gu and Liu, 2020).

Previous reviews in recent years have attempted to describe the mechanisms, impact size, and therapeutic implications of the radiation-induced neurocognitive decline in human and preclinical studies (Scoccianti et al., 2012; Pazzaglia et al., 2020; van Grinsven et al., 2021). In this review, we aim to: (1) identify the prediction models for radiation-induced neurocognitive decline, (2) abstract candidate and significant predictors, and (3) discuss the quality and applicability of available models in clinical practice.

## Methods

### Search Strategy

The MEDLINE database was searched systematically to identify relevant English articles published from inception to October 31, 2021. The search strategy consisted of a combination of subject mesh terms and truncation of free words. To identify the prediction model studies, a broader version of the previously validated search strategy published by Geersing et al. (2012) was combined with the terms related to “radiotherapy,” “brain,” and “neurocognitive impairments” (full search string provided in Supplementary Table 1). In addition, a manual search was conducted on references of the included articles. This review was carried out in accordance with the Preferred Reporting Items for Systematic Reviews and Meta-Analyses (PRISMA).

### Selection of Eligible Studies

Studies were included if they reported the development or external validation of at least one multivariable prediction model for specific or general neurocognitive deficit in adult patients who received either therapeutic or prophylactic brain irradiation for primary or metastatic brain tumors. Two independent reviewers (FT and DD) performed the title/abstract and full-text screening using the following exclusion criteria: (1) lack of model’s specifications, (2) no significant predictors in multivariate analysis, (3) univariate associations, (4) preclinical studies, (5) editorials, letters, conference abstracts, or non-original studies, or (6) no available full text. Disagreement between reviewers was resolved by consensus.

### Data Extraction

One reviewer extracted the data using a standard form designed according to the recommendation in the CHARMS statement (Moons et al., 2014). Extracted data included information about publication year, data source, sample size, characteristics of the study population (country, age, gender), type of primary tumor, treatment-related parameters (surgery, chemotherapy, irradiation technique, prophylactic intention), and outcome (definition, measuring instrument, and time of assessment). Moreover, the following information was extracted to assess the methodological considerations: modeling technique, event per predictor, candidate predictors, effect estimates of the included predictors, model’s intercept, and predictive performance measures (discrimination and calibration indices). A subsample of the extracted data (20%) was checked for correctness and completeness.

### Quality Assessment of Included Studies

The Prediction model Risk Of Bias ASsessment Tool (PROBAST) was used to assess the Risk Of Bias (ROB) of the identified prediction models (Moons et al., 2019). PROBAST uses 20 signaling questions to cover the four key aspects of the ROB in prediction studies (i.e., participants, predictors, outcome, and analysis). Each signaling question is answered as “yes,” “probably yes,” “no,” “probably no,” or “no information” and each domain is judged as “low risk,” “high risk,” or “unclear” based on the signaling questions in each domain. The overall ROB is rated as low risk (all domains are judged as low risk), high risk (at least one domain is judged as high risk), or unclear (at least one domain is judged as unclear and the remaining domains are judged as low risk). The applicability of prediction models to the review question was also judged as “low risk,” “high risk,” or “unclear” in terms of participants, predictors, and outcome.

## Results

### General Characteristics of Included Studies

**FIGURE 1:**
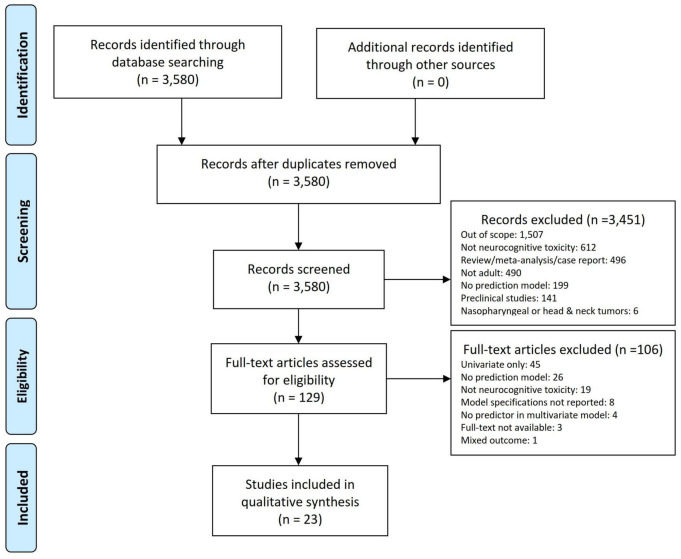
PRISMA flow diagram for inclusion and exclusion of studies.

The study selection process is shown in Figure 1. A total of 3,580 articles were retrieved. A total of 129 studies were retained for the full-text review after title/abstract screening. We further excluded 106 publications based on the exclusion criteria. Finally, 23 studies describing the development of prediction models were included, of which 16 (Gregor et al., 1996; Blay et al., 1998; Klein et al., 2002; Kaleita et al., 2004; van Beek et al., 2007; Wang et al., 2010; Starke et al., 2011; Gondi et al., 2012; Kangas et al., 2012; Chapman et al., 2016; Wong et al., 2019; Dutz et al., 2020; Gui et al., 2020; Tibbs et al., 2020; Langegård et al., 2021; Zamanipoor Najafabadi et al., 2021) studies included patients with primary and 7 (Wolfson et al., 2011; Gondi et al., 2013; Nakazaki and Kano, 2013; Chen et al., 2017; Yamamoto et al., 2017; Gui et al., 2019; Brown et al., 2020) with metastatic brain tumors. No external validation studies were identified.

As shown in Table 1, studies were published between 1996 and 2021, gradually increasing over the past few years. The prediction models were mainly developed in the United States (*n* = 13, 56.5%) (Kaleita et al., 2004; Wang et al., 2010; Starke et al., 2011; Wolfson et al., 2011; Gondi et al., 2012, 2013; Chapman et al., 2016; Chen et al., 2017; Gui et al., 2019, 2020; Wong et al., 2019; Brown et al., 2020; Tibbs et al., 2020), Netherlands (*n* = 3, 13%) (Klein et al., 2002; van Beek et al., 2007; Zamanipoor Najafabadi et al., 2021), and Japan (*n* = 2, 8.7%) (Nakazaki and Kano, 2013; Yamamoto et al., 2017). The median age of the study samples was 55 (IQR = 48–61) with median 54% (IQR = 45–60%) male gender. Only one study included elderly (age 70–79) and very elderly patients (age ≥ 80) with brain metastasis (Chen et al., 2017).

**TABLE 1:** Characteristics of the prediction model studies for radiation-induced neurocognitive decline in patients with primary or secondary brain tumors.

Study	Year	Country	Sample size	Primary tumor type	Outcome	Follow-up	Coefficient	Prediction equation	Model evaluation
**Primary brain or head and neck tumors**									
Gregor et al., 1996	1996	UK	30	Gliomas	NART, WAIS	>4 years	OR	(WBRT vs. Focused RT × 7.1)*	–
Blay et al., 1998	1998	France	226	Cerebral lymphomas	Neuroimaging	76 months	RR	(RT + chemotherapy × 11.5)*	–
Klein et al., 2002	2002	Netherlands	295	Gliomas	SCWT	12 years	RR	(Antiepileptic × 5.79) + (tumor lateralization × 5.3)*	–
Kaleita et al., 2004	2004	USA	79	Brain tumors	TMT-A	NR	Beta	3.932 + (frontal × 1.005) + (GBM × -0.812) + (Age 36–59 × -1.174)	–
van Beek et al., 2007	2007	Netherlands	81	Pituitary Adenoma	SF-36	10 years	Beta	(Radiotherapy × 0.56) + (male × 0.48) + (intact HPA axis × 0.57)*	–
Wang et al., 2010	2010	USA	299	Oligodendrogliomas	MMSE	6.9 year	Beta	(Assessment time × -0.013) + (KPS 80-100 × 2.724) + (age < 50 × 1.41)*	–
Starke et al., 2011	2011	USA	152	Meningioma	Neuroimaging	7 years	OR	(Tumor location clival/petrous × 4)*	–
Gondi et al., 2012	2012	USA	29	Brain tumors	WMS III WL	18 months	OR	(D40% of hippocampus > 7.3 Gy × 19.3)*	–
Kangas et al., 2012	2012	Australia	65	Brain tumors	FACT-G	3.5 months	Beta	(Malignant × -0.23) + (baseline PCL-S × -0.31) + (baseline FACT-G/Brain × 0.76) + (baseline POMS depression × -0.46)*	–
Chapman et al., 2016	2016	USA	27	Brain tumors	HVLT-PR	18 months	Beta	(Baseline HVLT-R × -0.62) + (frontotemporal × -2.19) + (age × -0.06)*	–
Wong et al., 2019	2019	USA	198	Brain tumors	DS, HVLT-R, COWA, TMT	6 months	OR	(Fatigue × 1.05)*	–
Gui et al., 2020	2020	USA	30	GBM	HVLT-R DR	36.1 months	Beta	(Mean dose to ipsilateral hippocampus × -0.064) + (mean dose to bilateral hippocampi × -0.084) + (mean dose to ipsilateral SVZ × -0.089) + (mean dose to bilateral SVZ × -0.13)*	–
Dutz et al., 2020	2020	Germany	62	Brain tumors	MoCA	2 years	Beta	-1.16 + (Left laterality × 2.37) + (cerebellum anterior V30Gy × -5.14) + (cerebellum anterior V40Gy × -6.85)	–
Tibbs et al., 2020	2020	USA	54	Brain tumors	DKEFS-TMT	12 months	Beta	(Beck anxiety inventories × -0.425)*	–
Zamanipoor Najafabadi et al., 2021	2021	Netherlands	190	Meningioma	DS, AVLT, CWFT, CST, MCT, SCWT	9 years	OR	(Age × 1.024) + (tumor size before last intervention × 1.022) + (second resection × 2.662) + (radiotherapy × 2.819) + (educational level × 0.359) + (years since diagnosis × 1.130)*	AUC: 0.78
Langegård et al., 2021	2021	Sweden	266	Brain tumors	QlQ-BN20	1–3 months	Beta	(Living alone × 3.97) + (SCQ > 4 points × 6.71)*	–
**Secondary brain tumors**									
Wolfson et al., 2011	2011	USA	75	Lung	HVLT, COWAT, TMT-A, TMT-B	25.3 months	OR	(Treatment type 2 Gy*18 × 8) + (treatment type 1.5 Gy*24 × 4.37) + (age × 1.12) + (education level ≤ High school × 2.96)*	–
Gondi et al., 2013	2013	USA	583	Lung	HVLT-R	12 months	OR	(No prophylactic cranial irradiation × 2.49) + (baseline impairment in HVLT-R × 3.33) + (age ≤ 60 × 2.52)*	–
Nakazaki and Kano, 2013	2013	Japan	76	Case-mix	MMSE	5.8 months	HR	(Volume of the largest metastasis × 1.102)*	–
Yamamoto et al., 2017	2017	Japan	1194	Case-mix	CTCAE v.3	46.3 months	HR	(Age < 65 × 1.455) + (large tumor with maximum diameter of largest tumor ≥ 1.6 cm × 0.375) + (neurologic symptoms × 0.413)*	–
Chen et al., 2017	2017	USA	119	Case-mix	RTOG	1–3 months	OR	(WBRT × 2.82)*	–
Gui et al., 2019	2019	USA	22	Lung	HVLT-R DR	24 months	Beta	(Absolute change in whole brain volume × 0.060) + (proportional change in whole brain volume × 0.79)*	–
Brown et al., 2020	2020	USA	518	Case-mix	HVLT-R	7.9 months	HR	(Age ≤ 61 × 0.635) + (HA-WBRT plus memantine × 0.745)*	–

AUC, Area Under the Receiver Operating Characteristic Curve; AVLT, Auditory Verbal Learning Test; COWAT, Controlled Oral Word Association Test; CST, Concept Shifting Test; CTCAE, Common Terminology Criteria for Adverse Events; CWFT, Categoric Word Fluency Test; D40%, equivalent dose in 2-Gy fractions (EQD2) assuming a/b = 2 Gy to 40% of the structure volume; DKEFS-TMT, Delis-Kaplan Executive Function System-Trail Making Test; DS, Digital Span; FACT-G, Functional Assessment of Cancer Therapy-General; GBM, Glioblastoma Multiforme; HA-WBRT, Hippocampal Avoidance-Whole-Brain Radiotherapy; HPA, Hypothalamic Pituitary Adrenal; HR, Hazard Ratio; HVLT-PR, Hopkins Verbal Learning Test-Percent Retained; HVLT-R, Hopkins Verbal Learning Test-Revised; HVLT-R DR, HVLT-R Delayed Recall; HVLT-R IR, HVLT-R Immediate Recall; ICD-9-CM, International Classification of Diseases 9th Clinical Modification; KPS, Karnofsky Performance Scale; MCT, Memory Comparison Test; MMSE, Mini Mental Status Examination; MoCA, Montreal Cognitive Assessment; NART, National Adult Reading Test; NR, Not Reported; OR, Odds Ratio; PCL-S, Posttraumatic stress disorder Checklist-Stressor; POMS, Profile of Mood States; QLQ-BN20, Quality of Life Questionnaire-Brain Neoplasm20; RR, Relative Risk; RT, Radiotherapy; RTOG, Radiation Therapy Oncology Group; SCQ, Self-Administered Comorbidity Questionnaire; SCWT, Stroop color-word test; SF-36, Short Form 36 Health Survey Questionnaire; SVZ, Sub-Ventricular Zones; TMT-A, Trail Making Test; UK, United Kingdom; USA, United States of America; WAIS, Wechsler Adult Intelligence Scale; WBRT, Whole-Brain Radiotherapy; WMS III WL, Wechsler Memory Scale-III Word List. *The intercept of the model is not reported.

Studies with primary brain tumors included the following tumor types: glioma (*n* = 2) (Gregor et al., 1996; Klein et al., 2002), meningioma (*n* = 2) (Starke et al., 2011; Zamanipoor Najafabadi et al., 2021), cerebral lymphoma (*n* = 1) (Blay et al., 1998), glioblastoma (*n* = 1) (Gui et al., 2020), oligodendroglioma (*n* = 1) (Wang et al., 2010), and pituitary adenoma (*n* = 1) (van Beek et al., 2007). Moreover, eight studies included the patients with different types of primary brain tumors (Kaleita et al., 2004; Gondi et al., 2012; Kangas et al., 2012; Chapman et al., 2016; Wong et al., 2019; Dutz et al., 2020; Tibbs et al., 2020; Langegård et al., 2021). The primary tumor site of the patients with metastatic brain cancer was: lung (*n* = 7), breast (*n* = 3), gastrointestinal (*n* = 3), kidney (*n* = 3), and skin (*n* = 2). Three studies included the patients who underwent prophylactic cranial irradiation (Wolfson et al., 2011; Gondi et al., 2013; Gui et al., 2019). Following irradiation techniques were used: intensity modulated radiotherapy (*n* = 4) (Chapman et al., 2016; Brown et al., 2020; Gui et al., 2020; Tibbs et al., 2020), stereotactic radiotherapy (*n* = 4) (Gondi et al., 2012; Kangas et al., 2012; Chen et al., 2017; Yamamoto et al., 2017), proton beam therapy (*n* = 3) (Dutz et al., 2020; Tibbs et al., 2020; Langegård et al., 2021), and gamma knife radiosurgery (*n* = 2) (Starke et al., 2011; Nakazaki and Kano, 2013). Eleven studies included the patients who were treated with chemotherapy (Wang et al., 2010; Wolfson et al., 2011; Kangas et al., 2012; Gondi et al., 2013; Chapman et al., 2016; Gui et al., 2019, 2020; Brown et al., 2020; Dutz et al., 2020; Tibbs et al., 2020; Langegård et al., 2021).

Regarding the data source, retrospective cohort was the most popular study design used for nine studies (Gregor et al., 1996; Blay et al., 1998; van Beek et al., 2007; Kangas et al., 2012; Nakazaki and Kano, 2013; Chen et al., 2017; Dutz et al., 2020; Langegård et al., 2021; Zamanipoor Najafabadi et al., 2021), followed by prospective cohort (seven studies) (Klein et al., 2002; Kaleita et al., 2004; Starke et al., 2011; Gondi et al., 2012; Chapman et al., 2016; Yamamoto et al., 2017; Gui et al., 2020), and prospective trials (seven studies) (Wang et al., 2010; Wolfson et al., 2011; Gondi et al., 2013; Gui et al., 2019; Wong et al., 2019; Brown et al., 2020; Tibbs et al., 2020). The median sample size of the cohorts was 81 (IQR = 54–266) and the median incidence of neurocognitive toxicity for studies with binary outcome was 26% (IQR = 14–73%). Three regression analyses were applied for model development: linear regression in 11 studies (48%) (Klein et al., 2002; Kaleita et al., 2004; van Beek et al., 2007; Wang et al., 2010; Kangas et al., 2012; Chapman et al., 2016; Gui et al., 2019, 2020; Dutz et al., 2020; Tibbs et al., 2020; Langegård et al., 2021), logistic regression in eight studies (35%) (Gregor et al., 1996; Starke et al., 2011; Wolfson et al., 2011; Gondi et al., 2012, 2013; Chen et al., 2017; Wong et al., 2019; Zamanipoor Najafabadi et al., 2021), and Cox regression in four studies (17%) (Blay et al., 1998; Nakazaki and Kano, 2013; Yamamoto et al., 2017; Brown et al., 2020). The intercept of the model was reported in two studies (Kaleita et al., 2004; Dutz et al., 2020). Among all studies, only one study performed internal validation in terms of Area Under the receiver operating characteristic Curve (AUC = 0.78) (Zamanipoor Najafabadi et al., 2021).

### Variables in the Prediction Models

**FIGURE 2:**
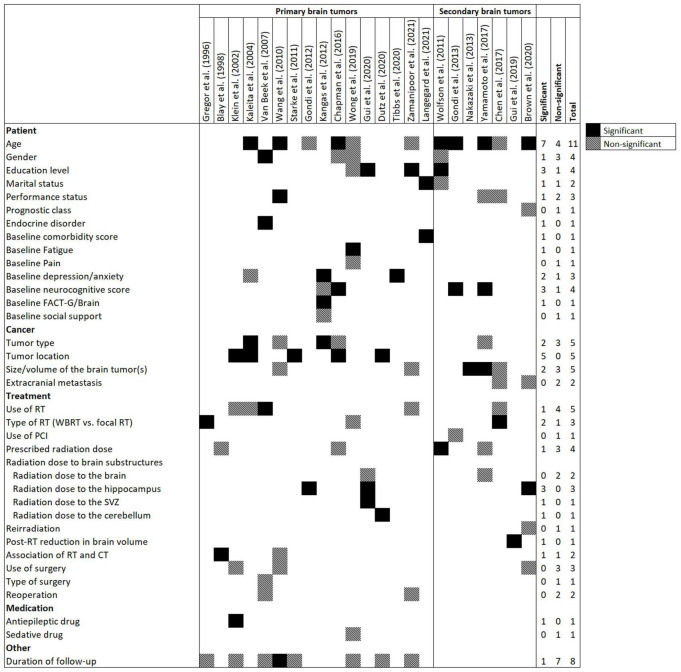
Frequency of candidate and significant predictors in prediction models for radiation-induced neurocognitive decline in patients with primary or secondary brain tumors. Abbreviations: CT, Chemotherapy; FACT-G, Functional Assessment of Cancer Therapy-General; PCI, prophylactic cranial irradiation; RT, Radiotherapy; SVZ, subventricular zone; WBRT, Whole brain RT.

Figure 2 presents candidate and significant predictors in the models. All variables of 23 prediction models were easily obtainable (*via* medical records, radiotherapy planning systems, and questionnaires), including socio-demographic, baseline comorbidities and neurocognitive functions, tumor-related variables, medication use history, and treatment-related parameters (radiotherapy, chemotherapy, and surgery). Age was the most common candidate predictor and was considered in 11 (48%) prediction models followed by duration of follow-up (*n* = 8, 35%), tumor type (*n* = 5, 22%), tumor location (*n* = 5, 22%), size/volume of brain tumor(s) (*n* = 5, 22%), and use of radiotherapy (*n* = 5, 22%).

The following variables were the most frequent significant predictors which remained in the models after multivariate analysis: age (*n* = 7, 30%), tumor location (*n* = 5, 22%), education level (*n* = 3, 13%), baseline neurocognitive score (*n* = 3, 13%), and radiation dose to the hippocampus (*n* = 3, 13%). The median number of significant predictors in prediction models was 2 (IQR = 1–3).

While radiation dose to the whole brain was removed from multivariate analysis in two prediction models (Yamamoto et al., 2017; Gui et al., 2020), dose to brain substructures, including hippocampus (*n* = 3) (Gondi et al., 2012; Brown et al., 2020; Gui et al., 2020), subventricular zone (*n* = 1) (Gui et al., 2020), and cerebellum (*n* = 1) (Dutz et al., 2020) remained significant in the prediction models.

### Outcome Assessment

The following tests were the most common instruments used for measuring the neurocognitive decline: Hopkins Verbal Learning Test (HVLT) (*n* = 7, 30%) (Wolfson et al., 2011; Gondi et al., 2013; Chapman et al., 2016; Gui et al., 2019, 2020; Wong et al., 2019; Brown et al., 2020), Trail Making Test (TMT) (*n* = 4, 17%) (Kaleita et al., 2004; Wolfson et al., 2011; Wong et al., 2019; Tibbs et al., 2020), Controlled Oral Word Association (COWA) (*n* = 2, 9%) (Wolfson et al., 2011; Wong et al., 2019), Digital Span (DS) (*n* = 2, 9%) (Wong et al., 2019; Zamanipoor Najafabadi et al., 2021), and Mini-Mental State Examination (MMSE) (*n* = 2, 9%) (Wang et al., 2010; Nakazaki and Kano, 2013). Three studies (13%) assessed the acute neurocognitive decline within the first 3 months after radiotherapy (Kangas et al., 2012; Chen et al., 2017; Langegård et al., 2021). The remaining studies assessed long-term neurocognitive side effects with a minimum of 6 months and maximum of 12 years duration of follow-up.

### Risk of Bias and Applicability

**FIGURE 3:**
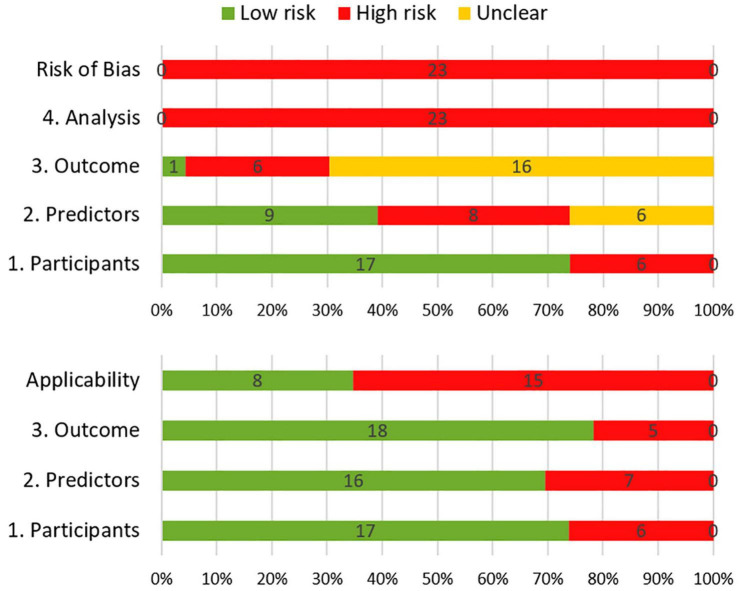
Summary of risk of bias (top) and applicability (bottom) according to the Prediction model Risk Of Bias ASsessment.

The results of the risk of bias and applicability assessment are shown in Table 2 and Figure 3. All models were judged to have a high risk of bias. The most common concerning issues were seen in analysis (domain 4), including lack of model validation and inappropriate or lack of handling missing data. Several models (*n* = 16, 70%) also had an unclear risk of bias in outcome assessment (domain 3) (Gregor et al., 1996; Klein et al., 2002; van Beek et al., 2007; Wolfson et al., 2011; Kangas et al., 2012; Nakazaki and Kano, 2013; Chen et al., 2017; Yamamoto et al., 2017; Gui et al., 2019, 2020; Wong et al., 2019; Brown et al., 2020; Dutz et al., 2020; Tibbs et al., 2020; Langegård et al., 2021; Zamanipoor Najafabadi et al., 2021). This was due to lack of information on outcome assessment without knowledge of predictors. Detailed ratings for underlying signaling questions are provided in Supplementary Table 2.

**TABLE 2:** Quality assessment for risk of bias and applicability concern of the included prediction models.

	Study	ROB				Applicability			Overall	
		Participants	Predictors	Outcome	Analysis	Participants	Predictors	Outcome	ROB	Applicability
Primary brain tumors	Gregor et al., 1996	–	+	?	–	–	+	+	–	–
	Blay et al., 1998	–	–	–	–	+	+	–	–	–
	Klein et al., 2002	+	+	?	–	+	+	+	–	+
	Kaleita et al., 2004	+	+	+	–	+	–	+	–	–
	van Beek et al., 2007	+	+	?	–	+	–	+	–	–
	Wang et al., 2010	+	?	–	–	+	–	+	–	–
	Starke et al., 2011	–	?	–	–	+	+	–	–	–
	Gondi et al., 2012	+	–	–	–	–	+	+	–	–
	Kangas et al., 2012	+	?	?	–	+	–	–	–	–
	Chapman et al., 2016	–	–	–	–	–	+	+	–	–
	Wong et al., 2019	+	?	?	–	+	+	+	–	+
	Gui et al., 2020	+	+	?	–	+	+	+	–	+
	Dutz et al., 2020	+	–	?	–	–	+	+	–	–
	Tibbs et al., 2020	+	–	?	–	+	–	+	–	–
	Zamanipoor Najafabadi et al., 2021	+	–	?	–	+	+	+	–	+
	Langegård et al., 2021	–	+	?	–	–	+	–	–	–
Secondary brain tumors	Wolfson et al., 2011	+	+	?	–	+	+	+	–	+
	Gondi et al., 2013	+	+	–	–	+	+	+	–	+
	Nakazaki and Kano, 2013	+	?	?	–	+	+	+	–	+
	Yamamoto et al., 2017	–	?	?	–	+	+	+	–	+
	Chen et al., 2017	+	+	?	–	–	+	–	–	–
	Gui et al., 2019	+	–	?	–	+	–	+	–	–
	Brown et al., 2020	+	–	?	–	+	–	+		–

*ROB, Risk of bias.*

*+ Indicates low ROB/low concern regarding applicability.*

*– Indicates high ROB/high concern regarding applicability.*

*? Indicates unclear ROB/unclear concern regarding applicability.*

Eight models received a low score for concerns about applicability, which indicates that they are well aligned with the review question (Klein et al., 2002; Wolfson et al., 2011; Gondi et al., 2013; Nakazaki and Kano, 2013; Yamamoto et al., 2017; Wong et al., 2019; Gui et al., 2020; Zamanipoor Najafabadi et al., 2021). The remaining models had high score for concerns about applicability. This was mostly due to concerns about applicability of the participants (domain 1), which reflects the use of ungeneralizable patient populations (e.g., secondary analysis of clinical trials).

## Discussion

This review summarized and evaluated 23 identified prediction models for neurocognitive decline after radiotherapy in patients with primary or metastatic brain tumors. The following risk factors were entered into at least two prediction models: age, tumor location, radiation dose to hippocampus, education level, baseline neurocognitive score, tumor type, size/volume of brain tumor(s), baseline depression/anxiety score, and type of radiotherapy (whole brain vs. focal). Although, many scholars have put substantial effort in developing prediction models for radiation-induced neurocognitive decline, the overall results are unsatisfactory. According to PROBAST, none of the models were judged to be at low risk of bias mainly due to limitations in modeling methodology.

The plethora of instruments measuring neurocognitive function is heartening. However, in the field of machine learning this negatively affects the comparability and reusability of the prediction models. There are a variety of aspects regarding the domains of instrument, how they are measured, and when specific neurocognitive functions are elicited (Cullen et al., 2007). This review clearly shows the gap in measuring the neurocognitive outcomes. Developing cognitive assessment instruments taking into account the peculiar traits of the different brain tumors and radiation modalities accompanied by their administration protocol would be beneficial toward developing a reliable prediction model.

A small number of significant predictors in prediction models (median = 2) as well as the exclusion of four studies due to lack of significant predictors in multivariate analysis may imply that researchers need to follow a more systematic method for predictor selection before modeling. Although there is no recommended approach for selecting candidate predictors, using existing data in the literature in addition to *a priori* knowledge of experts solicited from focus group discussions can be a solution to consider more predictive risk factors. The identified predictors in this review can be used as a potential set of predictors in future models.

Recent studies have documented deleterious associations between radiation dose to brain substructures and neurocognitive score in both pediatric and adult patients (Toussaint et al., 2019; Haldbo-Classen et al., 2020; Acharya et al., 2021; Eekers et al., 2021; Rodríguez de Dios et al., 2021). This is in line with the significant predictive power of radiation dose to the hippocampus, subventricular zone, and cerebellum in the available prediction models. This may provide important information about the radiation tolerance of the sub-volumes. In particular, it has been shown that equivalent doses of 2 Gy fractions to 40% of the hippocampus greater than 7.3 Gy is implicated in memory and learning impairments (Gondi et al., 2010, 2012). Although current evidence on region-specific neurocognitive decline is limited, it is potentially an interesting trend for future model development studies.

In terms of the geographical distribution of the prediction models, all models were developed in countries with high human development index where early detection rate is likely higher than in developing countries (Khazaei et al., 2020). Prediction models tailored to the population in less developed countries are needed before generalization and applications in clinical use.

About 26, 35, and 26% of the models had a high ROB in the participant, predictor, and outcome domains, respectively. However, high ROB in the analysis domain was observed in all prediction models. Two severe deficiencies in statistical analysis were rated as high risk in the majority of studies. The first deficiency was a lack of performance assessment. Prior to applying any of these prediction models into clinical practice, clinicians need to carefully consider the predictive performance of the models in different populations. Use in clinical practice can only be considered if the performance in the local clinical population is satisfactory. The second deficiency was lack of information on handling missing data. The majority of studies did not describe the method they used to manage missing data (removing subjects, single, or multiple imputation).

About 70% (*n* = 16) of the models used easily obtainable predictors, which would increase their applicability to clinical practice. It is reasonable that a combination of biomarkers and baseline neurocognitive scores would improve the predictive performance of the prediction models. However, prediction models including these variables were identified as high risk in applicability since these predictors are not routinely measured in daily practice. Another high concern regarding the applicability was due to an inappropriate data source. Developing a prediction model using data which have been collected during a clinical trial may not be generalizable to the intended target population.

The following limitations should be declared: First, differences between the included studies in terms of modality of treatment, type of brain tumor, and outcome assessment should be taken into account when interpreting the results from this study. Second, only English studies were included. Third, studies in non-peer reviewed literature (e.g., conference proceedings or research reports) were not considered. Fourth, quantitative synthesis of the effect estimates was not conducted due to the heterogeneity of outcomes.

In conclusion, 23 prediction models are available to estimate the risk of neurocognitive decline after radiotherapy in patients with primary or secondary brain tumors. The models present substantial heterogeneity in terms of outcome assessment. Moreover, the existing models were judged to have a relatively high risk of bias, with the leading limitation of lacking internal/external validation and also deficiencies in the statistical methodology for model development. For future studies it is important to carefully choose a set of candidate predictors including radiation dose to uniformly delineated brain substructures.

## Data Availability Statement

The original contributions presented in the study are included in the article/Supplementary Material, further inquiries can be directed to the corresponding author.

## Author Contributions

FT and DD performed the material preparation, data extraction, and analysis. CZ, JD, MA, AD, WV, and DE performed the interpretation of the results. AT performed the supervision. FT wrote the first draft of the manuscript. All authors contributed to the study conception and design, commented on previous versions of the manuscript, and read and approved the final manuscript.

## Conflict of Interest

The authors declare that the research was conducted in the absence of any commercial or financial relationships that could be construed as a potential conflict of interest.

## Publisher’s Note

All claims expressed in this article are solely those of the authors and do not necessarily represent those of their affiliated organizations, or those of the publisher, the editors and the reviewers. Any product that may be evaluated in this article, or claim that may be made by its manufacturer, is not guaranteed or endorsed by the publisher.
