# Supplementary material for: Prediction Models for Radiation-Induced Neurocognitive Decline in Adult Patients With Primary or Secondary Brain Tumors: A Systematic Review
Source: Front Psychol. 2022 Mar 31;13:853472. doi: 10.3389/fpsyg.2022.853472 (PMC9009149; doi:10.3389/fpsyg.2022.853472)
Supplement: Supplementary file 1 [file Data_Sheet_1.PDF]

Supplementary Material S1. Search strategy to identify the prediction models for radiation-induced neurocognitive decline in patients with primary or secondary brain tumors

	Domain	Results	Search Term
1	Radiotherapy	791,915	Radiotherapy[mh] OR Radiat*[tiab] OR Radiotherap*[tiab] OR Irradiat*[tiab] OR Radiosurg*[tiab]
2	Brain	2,294,669	Brain*[tiab] OR Brain[mh] OR Cerebr*[tiab] OR Crani*[tiab] OR Hemispher*[tiab] OR Inter-crani*[tiab] OR Intercrani*[tiab] OR Intracrani*[tiab] OR Intra-crani*[tiab] OR Skull*[tiab] OR Brainstem[tiab] OR "Posterior Fossa"[tiab]
3	Prediction Models	6,449,509	(Validat* OR Predict* OR Rule* OR Prognos* OR Forecast* OR Multivariate OR Univariate) OR (Predict* AND (Outcome* OR Risk* OR Model*)) OR ((History OR Variable* OR Criteria OR Scor* OR Characteristic* OR Finding* OR Factor*) AND (Predict* OR Model* OR Decision* OR Identif* OR Prognos*)) OR (Decision* AND (Model* OR Clinical* OR Logistic Models[mh])) OR (Prognostic AND (History OR Variable* OR Criteria OR Scor* OR Characteristic* OR Finding* OR Factor* OR Model*)) OR Artificial Intelligence[mh] OR Deep Learning[mh] OR Machine Learning[mh] OR Support Vector Machine[mh] OR Decision Trees[mh] OR Proportional Hazards Models[mh] OR Neural Network[mh] OR "Random Forest*" OR "Naive Bayes" OR "Bayes Classification" OR "Bayesian Network*" OR "Logit Model*" OR "Probit Model*" OR "Artificial Intelligence" OR "Deep Learning*" OR "Machine Learning*" OR "Support Vector Machine*" OR "Decision Tree*" OR "Cox Hazard" OR "Cox Proportional Hazard*" OR "Cox Regression" OR "Neural Network*"
4	Neurocognitive Impairment	4,134,805	"Neurocognitive Disorders"[mh] OR "Cognitive Dysfunction"[mh] OR "Cognition Disorders"[mh] OR "Memory Disorders"[mh] OR "Mental Disorders"[mh] OR Amnesia[mh] OR Depression[mh] OR Fatigue[mh] OR Dementia[mh] OR Delirium[mh] OR Neuropsychology[mh] OR Attention[mh] OR Memory[mh] OR "Executive Function"[mh] OR "Problem Solving"[mh] OR "Verbal Learning"[mh] OR "Mental Status and Dementia Tests"[mh] OR "Psychological Tests"[mh] OR "Neuropsychological Tests"[mh] OR "Memory and Learning Tests"[mh] OR "Speech Articulation Tests"[mh] OR "Word Association Tests"[mh] OR "Neurologic Examination"[mh] OR "Wechsler Memory Scale"[mh] OR "Trail Making Test"[mh] OR Hopkins Verbal Learning Test[tiab] OR HVLT[tiab] OR Hopkins Verbal Learning Test Revised[tiab] OR HVLT-R[tiab] OR COWA[tiab] OR Controlled Oral Word Association[tiab] OR Pegboard[tiab] OR Digit span[tiab] OR Digit symbol[tiab] OR Stroop[tiab] OR Neurocognit*[tiab] OR Cognit*[tiab] OR Neuropsycholog*[tiab] OR Neurotoxicit*[tiab] OR Intellectual[tiab] OR Memry[tiab] OR Mental[tiab] OR Amnesia[tiab] OR Dementia[tiab] OR Attention[tiab] OR Executive Function[tiab] OR Problem Solving[tiab] OR Verbal Learning[tiab] OR Word Association Tests[tiab] OR Epileps*[tiab] OR Seizure[tiab] OR Gait[tiab] OR Dysphasia[tiab] OR Headache[tiab] OR Delirium[tiab] OR Concentration[tiab]
**5**	**Final term**	**3,817**	**#1 AND #2 AND #3 AND #4**
**6**	**English filter**	**3,580**	

Table S2. Answers to PROBAST signaling questions for the model development studies for radiation-induced neurocognitive decline in patients with primary or secondary brain tumors

Study	ROB 1.1	ROB 1.2	ROB 2.1	ROB 2.2	ROB 2.3	ROB 3.1	ROB 3.2	ROB 3.3	ROB 3.4	ROB 3.5	ROB 3.6	ROB 4.1	ROB 4.2	ROB 4.3	ROB 4.4	ROB 4.5	ROB 4.6	ROB 4.7	ROB 4.8	ROB 4.9
Gregor et al. (1996)	PY	PN	PY	Y	Y	Y	Y	Y	Y	NI	Y	NI	Y	NI	PN	N	PY	N	N	Y
Blay et al. (1998)	PN	PY	N	Y	Y	PN	PN	Y	PY	NI	Y	Y	N	NI	PN	N	PN	N	N	Y
Klein et al. (2002)	Y	PY	PY	Y	Y	Y	Y	Y	PY	NI	Y	NI	Y	N	N	N	Y	N	N	Y
Kaleita et al. (2004)	Y	PY	PY	Y	PY	Y	Y	Y	Y	PY	PY	NI	N	NI	PN	PY	PY	N	N	Y
Van Beek et al. (2007)	PY	PY	PY	Y	Y	PY	Y	Y	Y	NI	Y	NI	PY	NI	N	PY	PN	N	N	Y
Wang et al. (2010)	PY	Y	NI	PY	Y	Y	Y	Y	PN	NI	PN	NI	N	PN	PN	Y	Y	N	N	Y
Starke et al. (2011)	PN	Y	NI	PY	Y	PY	PN	Y	PY	NI	PY	PY	Y	PN	N	N	PY	N	N	Y
Wolfson et al. (2011)	PY	Y	PY	PY	Y	Y	Y	Y	Y	NI	Y	PY	N	PY	N	PY	PN	N	N	Y
Gondi et al. (2012)	Y	PN	NI	NI	PY	Y	Y	Y	Y	PY	PY	N	Y	NI	N	N	Y	N	N	Y
Kangas et al. (2012)	Y	Y	Y	Y	PN	Y	Y	N	PY	PY	PN	NI	Y	PY	N	PY	PY	N	N	Y
Gondi et al. (2013)	Y	PY	Y	PY	PY	Y	Y	N	PY	NI	PN	NI	N	PN	N	N	NI	N	N	Y
Nakazaki et al. (2013)	PY	Y	Y	NI	PY	Y	Y	Y	PY	NI	PY	PY	PY	N	N	N	PN	N	N	Y
Chapman et al. (2016)	Y	PN	Y	PY	PN	Y	Y	N	PY	NI	PY	NI	Y	PN	N	N	PN	N	N	Y
Yamamoto et al. (2017)	Y	PY	Y	PY	PY	Y	Y	PY	PY	NI	PY	Y	N	PN	Y	N	Y	N	N	Y
Chen et al. (2017)	PN	PY	PY	NI	Y	Y	Y	Y	PY	NI	PY	NI	N	PN	N	PY	PY	N	N	Y
Gui et al. (2019)	Y	PY	Y	PY	PN	Y	Y	Y	PY	NI	PY	NI	Y	PY	N	N	PN	N	N	Y
Wong et al. (2019)	Y	PY	PY	NI	PY	Y	Y	PY	PY	NI	PY	Y	PN	PY	Y	N	PY	N	N	Y
Gui et al. (2020)	PY	PY	NI	NI	PN	Y	Y	Y	PY	NI	PY	NI	Y	PN	N	PY	PN	N	N	Y
Brown et al. (2020)	Y	PY	Y	Y	PY	Y	Y	Y	PY	NI	PY	NI	N	PY	Y	PY	Y	N	N	Y
Dutz et al. (2020)	PY	PY	PY	PY	PN	Y	Y	Y	PY	NI	PY	NI	Y	PY	N	N	Y	N	N	Y
Tibbs et al. (2020)	PY	PY	PY	NI	PN	Y	Y	Y	PY	NI	PY	NI	PY	PY	N	PN	PN	N	N	Y
Zamanipoor et al. (2021)	Y	PY	NI	NI	Y	Y	Y	Y	PY	NI	Y	PY	Y	PY	N	N	PN	NI	N	Y
Langegard et al. (2021)	Y	Y	PN	PY	PY	PY	Y	Y	PY	NI	PY	NI	PN	PY	PY	N	PN	N	N	Y

Abbreviations: N, No; NI, No Information; PN, Probably No; Y, Yes; PY, Probably Yes; ROB, Risk of Bias.
